# Endometrial Glucose Transporters in Health and Disease

**DOI:** 10.3389/fcell.2021.703671

**Published:** 2021-09-06

**Authors:** Ivana Vrhovac Madunić, Valentina Karin-Kujundžić, Josip Madunić, Ida Marija Šola, Ljiljana Šerman

**Affiliations:** ^1^Molecular Toxicology Unit, Institute for Medical Research and Occupational Health, Zagreb, Croatia; ^2^Department of Biology, School of Medicine, University of Zagreb, Zagreb, Croatia; ^3^Centre of Excellence in Reproductive and Regenerative Medicine, University of Zagreb School of Medicine, Zagreb, Croatia; ^4^Biochemistry and Organic Analytical Chemistry Unit, Institute for Medical Research and Occupational Health, Zagreb, Croatia; ^5^Department of Gynecology and Obstetrics, Sisters of Charity University Hospital, Zagreb, Croatia

**Keywords:** GLUT, SLC2, sodium glucose transporters, SLC5, endometrial stromal cells, endometrial decidualization, GLUT signaling, GLUTs’ epigenetic regulation

## Abstract

Pregnancy loss is a frequent occurrence during the peri-implantation period, when there is high glucose demand for embryonic development and endometrial decidualization. Glucose is among the most essential uterine fluid components required for those processes. Numerous studies associate abnormal glucose metabolism in the endometrium with a higher risk of adverse pregnancy outcomes. The endometrium is incapable of synthesizing glucose, which thus must be delivered into the uterine lumen by *glucose transporters* (GLUTs) and/or the *sodium-dependent glucose transporter 1* (SGLT1). Among the 26 glucose transporters (14 GLUTs and 12 SGLTs) described, 10 (9 GLUTs and SGLT1) are expressed in rodents and 8 (7 GLUTs and SGLT1) in the human uterus. This review summarizes present knowledge on the most studied glucose transporters in the uterine endometrium (GLUT1, GLUT3, GLUT4, and GLUT8), whose data regarding function and regulation are still lacking. We present the recently discovered SGLT1 in the mouse and human endometrium, responsible for controlling glycogen accumulation essential for embryo implantation. Moreover, we describe the epigenetic regulation of endometrial GLUTs, as well as signaling pathways included in uterine GLUT’s expression. Further investigation of the GLUTs function in different endometrial cells is of high importance, as numerous glucose transporters are associated with infertility, polycystic ovary syndrome, and gestational diabetes.

## Introduction

Intrauterine or early pregnancy loss occurs due to fetal chromosomal abnormalities, irregular maternal hormone secretion/action, or inappropriate nutritional support of uterine endometrium or embryos (Dean, 2019). More than 50% of pregnancies are lost in humans, mostly before or during embryo implantation, when there is high glucose demand. Glucose, a major source of metabolic energy, is crucial for endometrial decidualization and embryonic development (Pantaleon and Kaye, 1998). When glucose enters uterine fibroblasts, it is metabolized *via* multiple pathways to support decidualization, which is an essential process of endometrial stromal cells (ESCs) to support pregnancy. In decidua, the Warburg metabolism, a mechanism of glucose-derived carbon metabolism, is increased in this scenario, providing ATP and lactate for cell proliferation (Zuo et al., 2015). Inhibition of the glucose metabolism *via* the pentose phosphate pathway with glucosamine or dehydroepiandrosterone leads to impaired decidualization in mice (Frolova and Moley, 2011a; Tsai et al., 2013). Diabetic rodents likewise exhibit impaired endometrial decidualization (Garris, 1988; Burke et al., 2007). Moreover, obesity in women is associated with changes in the endometrial microenvironment affecting endometrial function and embryo implantation (Antoniotti et al., 2018). However, high glucose concentrations (up to 17.5 mM) did not impact the decidualization of human endometrial stromal cells (hESCs) *in vitro*, (Frolova and Moley, 2011a) which could be attributed to short-term exposure.

High glucose concentrations can be toxic to embryos, suggesting that there is an optimal glucose requirement for pre-implantation and embryo survival (Diamond et al., 1991; Bermejo-Alvarez et al., 2012). Dysregulation of the hexosamine biosynthetic pathway and O-linked protein glycosylation are the underlying mechanisms of glucotoxicity (due to hyperglycemia) acting on early embryo development (Pantaleon et al., 2010).

Embryo implantation is a synchronized process between an activated blastocyst and a receptive endometrium requiring glucose concentration sustained within a narrow range to optimize endometrial decidualization and embryo development. Otherwise, too high, or too low glucose levels could severely impair its development (Dean, 2019).

The uterine endometrium is a highly active tissue demanding a constant supply of glucose. It comprises two functionally different layers. The basal layer, attached to the myometrium, is stable and is not shed during the menstrual cycle. In contrast, the functional layer below the luminal epithelium is transient, undergoing changes throughout the cycle. The endometrium is composed of stromal cells, luminal and glandular epithelial cells, and endothelial cells (Frolova and Moley, 2011b). The endometrial glucose metabolism is enhanced when the epithelium and stroma prepare for embryo implantation. Furthermore, it is also high during the differentiation of the functional layer into the decidua, which is responsible for nutritional and physical support for the developing conceptus (embryo and associated membranes) (von Wolff et al., 2003; Frolova et al., 2009; Kim and Moley, 2009; Frolova and Moley, 2011b; Mori et al., 2016). The importance of the glucose metabolism for a successful pregnancy is indicated by the glycogen storage in endometrial epithelial cells during the implantation period, more precisely in the mid-secretory phase (von Wolff et al., 2003).

The uterine endometrium and conceptus cannot carry out gluconeogenesis. The first step in glucose utilization is its uptake into cells. Transmembrane transport of glucose in mammalian cells occurs *via glucose transporters* (GLUTs, SLC2 family) or *sodium-glucose linked transporters* (SGLTs, SLC5 family). GLUTs mediate glucose transport *via* facilitative diffusion, while SGLTs do so *via* the secondary active transport driven by the electrochemical Na^+^-gradient across the membrane (Hediger and Rhoads, 1994; Uldry and Thorens, 2004; Wright et al., 2011; Mueckler and Thorens, 2013; Wright, 2013). Thus far, 26 different glucose transporters, including 14 GLUTs and 12 SGLTs, have been described in humans and rodents, and their functions were proposed (Sabolic et al., 2012; Mueckler and Thorens, 2013; Wright, 2013; Vrhovac et al., 2015; Salker et al., 2017; Sharma et al., 2017; Vrhovac Madunic et al., 2017; Koepsell, 2020). The physiological function of numerous GLUTs relies on glucose as the main circulating fuel. Moreover, there is need for different cell type-specific glucose transporters with a variety of kinetic and regulatory properties (Mueckler and Thorens, 2013; Wright, 2013), as each GLUT isoform regulates glucose metabolism, gene expression, and differentiation or (patho) physiological conditions in different cell types (Vrhovac et al., 2014).

One or more GLUTs exist in every cell type, GLUT1–4 being the most studied forms. GLUT1 is expressed ubiquitously, and it is responsible for the basal uptake and glucose storage in all cells. GLUT2 is found in the liver, kidney, and small intestine, and it is associated with the insulin-dependent glucose uptake (Mueckler and Thorens, 2013). The high-affinity transporter GLUT3 is abundant in the brain, testes, placenta, and other organs with an intensive glucose metabolism (Maher et al., 1992; Burant and Davidson, 1994; Boileau et al., 1995; Hauguel-de Mouzon et al., 1997). GLUT4 is an insulin-dependent transporter that regulates fast glucose uptake into skeletal and heart muscle cells, adipocytes, and placental cells (Bell et al., 1990; Mueckler, 1990; Xing et al., 1998). GLUT8 is responsible for insulin-stimulated glucose uptake in the blastocyst (Carayannopoulos et al., 2000; Doege et al., 2000; Pinto et al., 2002). Different GLUTs are upregulated in cancer and other diseases, particularly GLUT1 and GLUT3, and their inhibitors have been proposed as novel approaches for treatments (Reckzeh et al., 2020).

SGLTs are responsible for complementing insulin and glucagon in the regulation of glucose homeostasis (Wright et al., 2011; Wright, 2013). The founding member of the SLC5 family, SGLT2, is a kidney-specific transporter found in healthy experimental animals and humans (Chen et al., 2010; Sabolic et al., 2012). SGLT2 and dual SGLT1/SGLT2 inhibitors have already been used in the treatment of diabetes (Vallon, 2015; Ghezzi et al., 2018). An overexpression of both transporters has been detected in diabetes and numerous cancer types (Scafoglio et al., 2015; Vallon, 2015; Koepsell, 2017; Vrhovac Madunic et al., 2018; Otto et al., 2020; Wright, 2020), making them a new target for cancer therapy.

Among these SGLTs, SGLT1, and SGLT2 have been frequently investigated, as they play key roles in the transport of glucose and sodium across the brush border membrane of intestinal and renal cells.

Data on the expression and function of endometrial GLUTs are very limited. According to the literature, nine GLUTs have been described in the rodent and seven in the human uterus. The only finding of the high-affinity Na^+^-coupled glucose transporter in the endometrium is that of SGLT1 in mouse and human epithelial cells (Salker et al., 2017).

**FIGURE 1:**
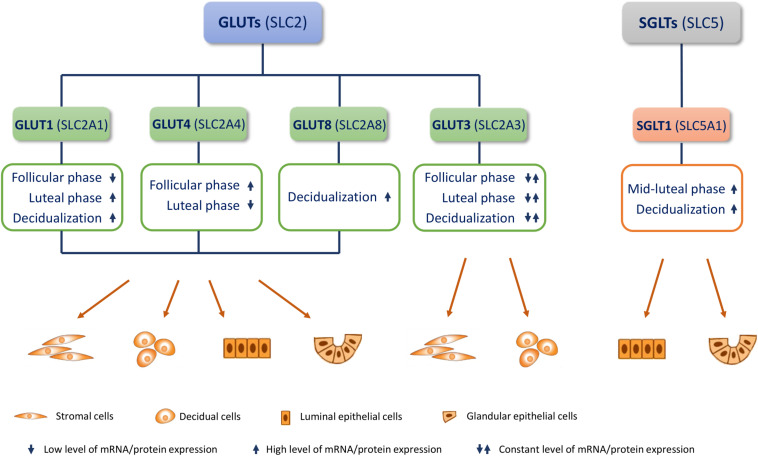
Endometrial glucose transporters (GLUTs) and sodium-glucose transporter 1 (SGLT1). Most studied GLUTs and SGLTs in different cell types of the endometrium and changes in their mRNA/protein expression in the menstrual cycle (follicular/proliferative phase and luteal/secretory phase) and during decidualization, are presented.

Here, we summarize present knowledge on the most studied glucose transporters in the endometrium: GLUT1, GLUT3, GLUT4, GLUT8, and the recently discovered SGLT1 (Figure 1). We also describe the function of GLUTs in uterine physiology (menstrual cycle and early pregnancy), their epigenetic regulation, cell signaling, and their role in infertility and polycystic ovary syndrome (PCOS).

The aim of this review is to broaden the knowledge about the endometrial role of GLUTs in healthy and diseased conditions.

### GLUT1

GLUT1 (*SLC2A1*) was the first facilitative glucose transporter to be discovered, having been purified from human erythrocytes (Kasahara and Hinkle, 1977) and cloned from HepG2 cells (Mueckler et al., 1985). GLUT1 is ubiquitously expressed in all organs, ranging from endothelial cells of the blood-brain barrier to fetal tissues; however, it is most abundant in erythrocytes, where it increases the cells’ glucose carrying capacity (Mueckler and Thorens, 2013). Besides glucose, it transports other sugars including mannose, galactose, glucosamine, and reduced ascorbate (Carruthers et al., 2009; Mueckler and Thorens, 2013). Several research groups reported GLUT1 expression in the placenta (Fukumoto et al., 1988; Bell et al., 1990; Farell et al., 1992; Takata et al., 1992; Jansson et al., 1993; Clarson et al., 1997). In different cells and tissues, GLUT1 is regulated by glucose concentration, insulin, and growth factors (Mueckler and Thorens, 2013).

GLUT1 was the first GLUT to be found in the endometrium; more precisely, its mRNA is abundant in ESCs of both human and rodents. High *GLUT1* levels have been detected in decidua during pregnancy, implying the role of *GLUT1* in maintaining pregnancy and fetal development (Sakata et al., 1995; Yamaguchi et al., 1996; Frolova et al., 2009). Furthermore, it was shown that during decidualization, GLUT1 protein levels can increase up to 10 times and are accompanied by an increase in the glucose uptake *in vivo* (Frolova et al., 2009; Neff et al., 2020). GLUT1 upregulation has also been reported in decidualized hESCs (Tamura et al., 2014). In contrast, downregulated GLUT1 in ESCs lead to a reduced glucose uptake and suppressed decidualization (Huang et al., 2019). Accordingly, in cultured hESCs, the *GLUT1* knockdown impaired decidualization (Frolova and Moley, 2011a). A recent study reported that hESCs are able to survive the hypoxic environment by activating metabolic pathways *via* GLUTs, which could be relevant for the menstrual and implantation period (Kido et al., 2020).

In the human endometrium, *GLUT1* expression is upregulated during the peri-implantation period, and is higher during the secretory phase compared to the proliferative phase (Korgun et al., 2001; Strowitzki et al., 2001; Zhang et al., 2020). In contrast, no difference was observed in the expression of *GLUT3* in different phases. This result is consistent with immunohistochemical analysis (Zhang et al., 2020). An adequate glucose metabolism is crucial for endometrial differentiation and decidualization processes. These processes are mainly mediated by progesterone (P4) and partially by 17β-estradiol (E2). *In vitro* studies demonstrated that GLUT1 is upregulated by P4 in murine ESCs, and furthermore that 17β-estradiol E2 reverses the effect of P4, bringing GLUT1 expression back to its basic level (Frolova et al., 2009). Although this finding indicates the opposite effect of E2 and P4 on GLUT1, both hormones are present at the time of implantation, indicating their interplay in the regulation of GLUT1. A recent study confirmed these findings *in vivo*, showing that P4 was responsible for the upregulation of GLUT1 protein in the murine uterus (Zhang et al., 2020). In women with idiopathic infertility, GLUT1 is significantly reduced, suggesting its enrolment in the regulation of endometrial function (von Wolff et al., 2003). Further, a *GLUT1* knockdown resulted in failed embryo implantation *in vivo*, indicating that GLUT1 silencing affects the endometrium, rather than the embryo (Zhang et al., 2020). These findings propose that pregnancy failure may be associated to a lower GLUT1-mediated glucose metabolism, resulting in reduced endometrial receptivity.

A recent *in vitro* study showed that high insulin concentrations downregulate the GLUT1 gene and protein expression, which is accompanied by a slight reduction in the glucose uptake in decidualizing hESCs (Jakson et al., 2020). Further clinical studies are necessary to determine the importance of insulin regulation in endometrial function and decidualization in patients with insulin resistance and hyperinsulinemia.

### GLUT3

GLUT3 (*SLC2A3*) is a high-affinity glucose transporter expressed in tissues with an intensive glucose metabolism, such as the brain, placenta, preimplantation embryos, sperm, and testes (Maher et al., 1992; Shepherd et al., 1992; Haber et al., 1993; Burant and Davidson, 1994; Boileau et al., 1995; Hauguel-de Mouzon et al., 1997; Pantaleon et al., 1997).

In the human endometrium, GLUT3 is expressed in the proliferative phase and decidua at a constant level throughout different menstrual stages or in early pregnancy (Hahn et al., 2001; von Wolff et al., 2003; Korgun et al., 2005). GLUT3 and GLUT1 are differentially expressed in utero-placental modulation. In mouse decidua, GLUT1 is upregulated after mid-pregnancy, whereas GLUT3 is slightly downregulated, indicating that this glucose transporter is not crucial after mid-pregnancy (Yamaguchi et al., 1996). This decrease was confirmed in rats, where GLUT3 is found in the uterine epithelium and stroma, and its expression increases through day 4 since gestation (Korgun et al., 2001). A correlation between placental and uterine GLUT3 expression with serum progesterone levels has been shown during early pregnancy (Ferre-Dolcet et al., 2018).

Studies using GLUT3 KO mice showed that GLUT3 is not essential for fertilization or implantation of blastocysts, but for the development of early post-implanted embryos. The deletion of *GLUT3* inhibits early embryonic development due to apoptosis of ectodermal cells shortly after implantation (Schmidt et al., 2009).

In summary, GLUT1 is a basal transporter that is responsible for constant glucose intake during the entire pregnancy, whereas GLUT3 is required for optimizing glucose uptake in the first trimester when maternal circulation through the placenta is not yet established. Putatively, GLUT3 may function as a “scavenger” glucose isoform, acting in circumstances when extracellular glucose levels are lower than the normal circulating ones (Brown et al., 2011). Therefore, GLUT3 may represent an important transporter for fetal supply in early gestation. However, further studies on GLUT3 expression and function in the endometrium are required.

### GLUT4

GLUT4 (*SLC2A4*) is an insulin-regulated transporter found in the heart, skeletal muscle, adipose tissue and brain (Bryant et al., 2002; McKinnon et al., 2014). Reversible translocation of GLUT4 to the cell surface, stimulated by insulin, leads to rapid glucose uptake into the cell (Pessin et al., 1999; Bryant et al., 2002). Several studies found muscle and fat cell insulin resistance to be associated with decreased GLUT4 expression and its impaired translocation (Dunaif, 1997; Shepherd and Kahn, 1999; Mueckler, 2001), contributing to insulin resistance and Type II diabetes (Bryant et al., 2002).

Data on GLUT4 in endometrial tissue are scarce and inconsistent. Endometrial GLUT4 was detected only in epithelial cells by some research groups (Mioni et al., 2012), while others found it in both stromal and epithelial cells (Cui et al., 2015). In contrast, some reported the absence of GLUT4 in endometrial tissue and stromal cells (von Wolff et al., 2003; Frolova and Moley, 2011a).

Insulin-dependent endometrial GLUT4 was found maximally expressed in the follicular phase of menstrual cycle, with a decline in the luteal phase (Mioni et al., 2012; Zhai et al., 2012; Cui et al., 2015). In contrast, GLUT1, which is an insulin independent transporter, was found maximally expressed in the luteal phase and mainly localized in the cytoplasm of stromal cells predetermined to transform into decidual cells (Mioni et al., 2012).

Although little is known about the insulin dependence of the endometrium, GLUT4 was found in endometrial epithelial cells (Mioni et al., 2004; Mozzanega et al., 2004; Fornes et al., 2010; Zhai et al., 2012). Moreover, Mioni et al. (2004) demonstrated that GLUT4 is dysregulated in the endometrium of PCOS patients. This is important, as PCOS is the most common metabolic and endocrine disorder affecting 5–10% of all women of reproductive age (Diamanti-Kandarakis, 2008). Chronic anovulation is one of its hallmarks, and about 75% of infertility due to anovulation is attributed to PCOS (Costello and Eden, 2003). Other important clinical manifestations of PCOS may also include obesity, insulin resistance (IR), and signs of androgen excess (Rotterdam ESHRE/ASRM-Sponsored PCOS Consensus Workshop Group, 2004).

Endometrial GLUT4 is significantly decreased in hyperinsulinemic women with PCOS, compared to those with PCOS but without IR. This implies a link between hyperinsulinism and GLUT4 downregulation. In women with PCOS and no IR, endometrial GLUT4 was significantly decreased only in obese women (Mioni et al., 2004). This is important, as IR is found in 50–70% of all women with PCOS (Sirmans and Pate, 2013), consequently leading to hyperinsulinism (Dunaif, 1997) in both obese and non-obese patients, with obesity aggravating IR (Maison et al., 2001). It is noteworthy that 30–50% patients with PCOS are obese (Sam, 2007; Barber et al., 2019). Hyperandrogenemia negatively affects glucose uptake in endometrial cells regulated by GLUT4 (Rosas et al., 2010; Zhang and Liao, 2010; Rivero et al., 2012; Li et al., 2015). However, one study reported no connection between the hyperandrogenic syndrome *per se* and GLUT4-regulated glucose uptake (Mioni et al., 2004). All these findings can possibly explain the reduced pregnancy rate in women with PCOS at only 30–40%, although their ovulation rate reaches up to 80% (Imani et al., 2002). Furthermore, they experience a higher rate of spontaneous and recurrent abortions (Giudice, 2006). It has been shown that hyperinsulinemia does not have a direct effect on LH secretion, and therefore does not cause the hypothalamic-pituitary-ovarian axis dysfunction in PCOS. However, *in vivo* and *in vitro* studies confirmed that insulin excess stimulates further androgen production in ovaries *via* insulin receptors on theca/interstitial ovarian stromal cells, therefore aggravating already increased ovarian androgen production in PCOS and lowering the hepatic sex-hormone binding globulin (Smith et al., 1987; Plymate et al., 1988; Nestler et al., 1998; Baillargeon, 2007; Rosenfield and Ehrmann, 2016).

IR in PCOS patients is commonly treated with antidiabetic drug metformin (1,1-dimethylbuguanide hydrochloride), which is known to improve insulin sensitivity and decrease insulin and androgen levels, thus promoting ovulation (Harborne et al., 2003; Lord et al., 2003). Endometrial GLUT4 is lower in PCOS than in non-PCOS patients. However, after 3 months of metformin treatment, *GLUT4* mRNA and protein expression is increased in the endometrium of PCOS patients (Zhai et al., 2012). Moreover, metformin directly restores endometrial function by increasing GLUT4 expression (Carvajal et al., 2013). In addition, metformin increases serum IGF-1 and glycodelin during the luteal phase (Jakubowicz et al., 2001; Palomba et al., 2006), which may contribute to an improved endometrial environment for the establishment and maintenance of pregnancy (Carvajal et al., 2013).

GLUT4 may be regulated by steroid hormones. Progesterone (P4) alone, and in combination with estradiol (E2), decreases endometrial GLUT4, suggesting that an abnormal hormonal secretion pattern, such as in PCOS, could disrupt normal GLUT4 expression (Cui et al., 2015).

The importance of GLUT4 in the endometrium is well established, although numerous studies were performed on only a small number of patients. Therefore, the elucidation of endometrial GLUT4 function and its molecular mechanisms in physiological and pathophysiological (such as PCOS) states is of high interest. Furthermore, due to its limited cellular expression and inducible nature, GLUT4 could be a desirable target for new non-hormone-based drugs (McKinnon et al., 2014).

### GLUT8

The GLUT8 (*SLC2A8*) transporter is mostly present in the brain and testes (Doege et al., 2000; Carosa et al., 2005), but is also detected in the uterus, ovary, and term placenta (Carayannopoulos et al., 2000; Ibberson et al., 2000; Limesand et al., 2004; Schmidt et al., 2009). It is localized in intracellular membranes of predominantly late endosomes and lysosomes, as well as in the ER and *trans*-Golgi (Stanirowski et al., 2017). The late-endosomal/lysosomal limiting membrane is the cellular hub for various metabolic reactions (e.g., mTORC1 recruitment to the lysosomal membrane, modulation of mitochondrial homeostasis, regulation of protein, and lipid content of the cellular membrane and membranes of various vesicles). Therefore, besides transporting glucose, GLUT8 may also function as a sensor for various metabolites in cellular metabolic homeostasis (Alexander et al., 2020). Some of the GLUT8 is cleaved into N-terminal and C-terminal peptides. Of its 12 *trans-*membrane domains, the cleavage occurs at domain 10, which happens to be the only one that is 100% conserved across the species. The full-length and N-terminal cleaved proteins are retained at the endosomal/lysosomal boundary, whereas the carboxy domain accumulates in a different population of vesicles and is unlikely to perform transport (Alexander et al., 2020). GLUT8 is one of several GLUT species that exists as several alternatively spliced mRNA variants, and there is few available data on its functionality. Alexander et al. (2020) concentrated on its expression in breast cancer and found that GLUT8 has three such variants: v1 (full-length), v2 (missing exon 9 and part of the C-terminus) and v3 (missing exon 2 and 3). In normal human tissue, by far the highest expressions of v1 and v3 mRNA variants are found in the testis, followed by liver and muscle, and only very little in the mammary gland, with the full-length version being predominant and v3 accounting for only about 5–10% of the total GLUT8 mRNA. The expression survey found that only the full-length GLUT8 variant is translated into stable, functional protein in both normal and tumor tissues (Alexander et al., 2020).

In the endometrium, GLUT8 can provide glucose required for protein glycosylation in the ER during decidualization (Frolova and Moley, 2011b). *GLUT8* is elevated in ESCs upon decidualization in mice and humans, whereas the protein expression is not increased (Frolova and Moley, 2011a). Mouse *Slc2a8-/-* mutants exhibited disordered decidualization, indicating that SLC2A8 plays an important role in this process (Adastra et al., 2012). Among other GLUTs, only *GLUT8* was found to be reduced in hESCs in a hypoxic environment. Recently, it was reported that suppressing GLUT8 under hypoxia might promote the preferential use of glucose for glycolysis (Kido et al., 2020). Hence, gaining an understanding of hESCs behavior in the hypoxic conditions of menstrual and implantation periods is of great importance.

GLUT8 is anchored at intracellular membranes, and it is not sensitive to insulin in most tissues, unlike certain other GLUT family members (e.g., GLUT4 and GLUT12). The blastocyst is an exception. Namely, it has been shown that in the blastocyst, GLUT8 translocates to the plasma membrane in response to insulin stimulation and imports glucose. Thus, it is considered critical for murine blastocyst survival (Carayannopoulos et al., 2000; Pinto et al., 2002; von Wolff et al., 2003).

### Sodium-Glucose Transporter 1

Sodium-glucose transporter 1 is primarily expressed in the small intestine and kidneys, but it is also found in the heart, liver, pancreas, brain, lungs, prostate, and uterus (Wright et al., 2004; Vallon et al., 2011; Gorboulev et al., 2012; Kashiwagi et al., 2015; Vrhovac et al., 2015; Salker et al., 2017; Sharma et al., 2017; Vrhovac Madunic et al., 2017).

The first report of SGLT1 expression and activity in murine and human endometrial epithelial cells showed that it is responsible for controlling glycogen accumulation essential for embryo implantation (Salker et al., 2017). Endometrial glycogen is associated with fertility. During peri-implantation and in early pregnancy, the increase in endometrial glycogen represents a highly important source of glucose. Glycogen production requires the cellular uptake of glucose, mostly by passive transporter GLUTs. Recently, another mechanism of accumulating glucose has been demonstrated *via* the secondary active transporter SGLT1, which can mediate glucose uptake even at low extracellular glucose concentrations (Salker et al., 2017). Glucose transport across the endometrium (at the estrus phase of the cycle) was detected in wild type, but not in *SGLT1*-knockout mice. The SGLT1 deficiency notably decreased the endometrial glycogen, litter size, and weight of the offspring. Moreover, SGLT1 was upregulated upon the decidualization of primary hESCs, which is crucial for embryo implantation. Furthermore, endometrial SGLT1 levels are significantly lower in women with recurrent pregnancy loss when compared to women with a healthy pregnancy. The study reveals a novel mechanism for adequate endometrial glycogen storage for pregnancy as a key factor for embryo implantation. Overall, endometrial SGLT1 deficiency in the human during implantation may lead to early pregnancy failure and obstetrical complications, including low fetal growth (Salker et al., 2017). These results suggest that caution is needed in the period of preconception and during pregnancy if SGLT1 inhibitors are considered for the treatment of diabetes (Vallon, 2015; Song et al., 2016).

### Signaling Pathways in GLUT Expression

One of the crucial signaling pathways in the glucose metabolism is the insulin pathway, with insulin acting as a main regulator of blood glucose concentration by increasing glucose uptake in muscles and fatty tissue, while inhibiting its production in liver (Saltiel and Kahn, 2001). The primary function of insulin is to maintain glucose homeostasis by translocating GLUT4 to the cell surface *via* activation of the PI3K/Akt pathway (Bryant et al., 2002). Maintaining the physiologically relevant narrow range of blood glucose concentration requires tight and rapid control of insulin signaling.

An essential step in successful pregnancy is decidualization of the human endometrium, a process consisting of many morphological changes and a functional differentiation of ESCs. This process depends on a complex interaction of transcription factors, cytokines, cell cycle regulators, and signaling pathways (Okada et al., 2018). Proliferation of the endometrium is induced by insulin through the insulin-like growth factor 1 (IGF-1), induced by estrogen in the proliferative phase (Strowitzki et al., 2006). A study by Nandi et al. (2010) reported that impaired insulin signaling affects female reproduction health through the hypothalamic-pituitary-gonadal axis, resulting in altered cycles and follicular development. Authors also observed unsuccessful pregnancies, increased calcification of placentae, and small embryos in insulin-resistant and hyperinsulinemic model mice. Other studies proposed that hyperandrogenism in the ovaries of PCOS patients could disrupt the insulin pathway (Zhang and Liao, 2010) and affect the function of the endometrium (Diamanti-Kandarakis and Papavassiliou, 2006). Furthermore, hyperinsulinism observed in PCOS patients lowers the concentration of circulating glycodelin (biomarker of endometrial function) and insulin-like growth factor-binding protein-1 (IGFBP-1), a feto-maternal interface adhesion factor. This contributes to early pregnancy loss due to diminished endometrial receptivity caused by decreased uterine vascularity and endometrial blood flow (Jakubowicz et al., 2001). A recent study showed that the deletion of ovary-specific insulin and/or IGF-1 receptor(s) caused infertility in mice as a consequence of impaired ovulation, luteal differentiation, uterine receptivity, and steroid hormone signaling (Sekulovski et al., 2020). Furthermore, dual receptor ablation compromised decidualization, reduced endometrial thickness, and inhibited embryo implantation. Authors further observed decreased activation of ERK/MAPK signaling (Sekulovski et al., 2021). Other pathological states, such as inflammation and obesity, cause insulin resistance by serine phosphorylation of the insulin receptor or insulin receptor substrate (IRS) proteins by TNF-α and PKCε (Hotamisligil, 1999). A subsequent decrease in tyrosine phosphorylation inhibits kinase activity of IR. A study by Bergman et al. (2019) suggested that IR and type 2 diabetes can be caused by reduced hepatic clearance of insulin. Samuel and Shulman (2012) reported ectopic lipid accumulation, and the activation of unfolded protein response likewise led to the development of IR.

Other signaling pathways have been linked to the regulation of GLUT expression. Models of artificial decidualization and delayed/activated implantation have demonstrated that the expression of murine uterine epithelial GLUT1, GLUT8, and GLUT9B are regulated by estradiol and progesterone through Akt/MAPK/PRKAA signaling (Kim and Moley, 2009). This result implies that the hormonal changes associated with early pregnancy affect GLUT expression and indicate an important role of uterine glucose utilization in endometrial decidualization, embryo implantation, and maintenance of pregnancy. Similarly, GLUT1 has been shown to be induced by progesterone-activated Hif1α and c-Myc *via* the PI3K/Akt signaling pathway during decidualization at the sites of murine embryo implantation. The corresponding increase in GLUT1 expression was one of the drivers of decidual glycolysis taking place under normoxic conditions, a phenomenon the authors termed Warburg-like glycolysis (Zuo et al., 2015). Another study analyzed the GLUT isoform expression in a PCOS-like model of uterine IR and hyperandrogenism (Zhang et al., 2016). Their results revealed both an increase and decrease in the mRNAs expression in rats treated with insulin. In contrast, most *GLUT* genes (including *GLUT4*) were downregulated in hyperandrogenic conditions. The authors suggested that chronic treatment with insulin and hCG causes changes in the GLUT expression due to altered PI3K/Akt and MAPK/ERK signaling pathways, which contributes to the initiation and development of local uterine IR. Another signaling pathway connecting GLUT expression and uterine decidualization is Notch signaling. *In vitro* and *in vivo* experiments demonstrated that inhibition of Notch signaling through endometrial loss of the RBPJ transcription factor leads to impaired decidualization in the murine uterus and in human endometrial fibroblasts associated with reduced progesterone receptor and GLUT1 expression (Strug et al., 2019).

### Epigenetic Regulation of Glucose Transporters

Knowledge about epigenetic regulation of endometrial GLUTs is limited, as there is almost no experimental data on the epigenetic regulation of these transporters in the endometrium. Epigenetic modifications, including histone modification, DNA methylation, and non-coding RNAs, play an important role in the decidualization of hESCs by influencing the expression of target genes (Sugino et al., 2016; Liu et al., 2020). A genome-wide analysis of histone modifications showed that the induction of decidualization increases H3K27ac and H3K4me3 signals in many proximal and distal promoter regions in the hESCs. These modified promoter regions are involved in the upregulation of gene expression that occurs during decidualization. Regions with increased H3K27ac and H3K4me3 signals are associated with the insulin signaling pathway, which may enhance glucose uptake and thereby contribute to decidualization (Tamura et al., 2014). It has also been suggested that the insulin signaling pathway contributes to decidualization through the increase of glucose uptake, and showed that glucose may regulate histone acetylation of gene promoters in decidualizing stromal cells (Jozaki et al., 2019). However, there are conflicting studies regarding the involvement of the insulin signaling pathway in glucose uptake during decidualization. Endometrial expression of GLUT4, the most important insulin-dependent glucose transporter, was higher in the proliferative than in the secretory phase of the menstrual cycle, which is in agreement with another study (Mioni et al., 2012; Cui et al., 2015). The increase in the endometrial insulin receptor expression was not coupled with a parallel increase of GLUT4, which significantly decreased from the proliferative to secretory phase, suggesting that insulin resistance can be achieved at the endometrial cell level in the secretory phase of the menstrual cycle (Mioni et al., 2012). The epigenetic regulation of endometrial GLUT4 expression is unclear. It has been demonstrated that the estrogen receptor β (ERβ) is recruited to the region of *Glut4* promoter containing CpG in island 1 (CpG11). The transcription of *Glut4* was markedly reduced in mouse embryonic fibroblasts lacking ERβ. The authors showed that ERβ protects the *Glut4* promoter from DNA methylation, which is involved in *Glut4* regulation (Rüegg et al., 2011). In the human endometrium, the ERβ mRNA level was highest during the proliferation phase and noticeably decreased with the progression of the secretory phase (Matsuzaki et al., 1999). Because GLUT4 levels decrease from the proliferative to the secretory phase (Mioni et al., 2012; Cui et al., 2015), ERβ could be responsible for mediating epigenetic events and thereby regulating endometrial *GLUT4* expression throughout the menstrual cycle.

Several miRNAs have been validated as direct inhibitors of *GLUT4* expression: miR-93-5p, miR-106b-5p, and miR-223-3p. These miRNAs are upregulated in adipose or muscle tissues of humans or animals with IR (Chen et al., 2013; Chuang et al., 2015; Zhou et al., 2016; Esteves et al., 2017). MiR-93-5p expression correlated negatively with *GLUT4* expression in subcutaneous adipose tissue of women with PCOS (Chen et al., 2013); however, this has not yet been established for endometrial tissue. Women with PCOS exhibited a lower level of *GLUT4* mRNA in their proliferative endometrium (Ujvari et al., 2014), which could impair glucose uptake in ESCs and thereby negatively affect decidualization. Whether the abovementioned miRNAs are involved in the regulation of endometrial *GLUT4* in PCOS and non-PCOS women would be an interesting research topic.

Moreover, progesterone-induced miR-152 regulates glucose concentration of uterine fluid by downregulating *Glut3* in the mouse endometrial epithelium, thus affecting early embryonic development and implantation (Nie et al., 2019). Apart from the miRNA already mentioned above, long non-coding RNA NICI could also regulate *GLUT3*, which is the case in different types of human cells. This regulation is mediated through transcriptional activation rather than post-transcriptional mechanisms. It was shown that hypoxia drives *GLUT3* expression through hypoxia-inducible transcription factor (HIF)-mediated induction of NICI expression (Lauer et al., 2020). Hypoxic conditions could occur during the menstrual and implantation period (Maybin and Critchley, 2015; Matsumoto et al., 2018; Kido et al., 2020). GLUT1 is important for the glycolytic metabolism of hESC in hypoxic environments (Kido et al., 2020). GLUT3 is also induced in an hypoxic environment (Mimura et al., 2012), but this has not yet been reported for endometrial GLUT3. In summary, long non-coding RNA NICI could induce endometrial *GLUT3* expression in hypoxic conditions, which could occur during the menstrual and implantation period.

Further studies are needed to explore the epigenetic modifications involved in the regulation of *GLUT* gene expression, thereby affecting glucose metabolism during decidualization.

## Conclusion and Future Perspectives

Several GLUTs and the recently detected SGLT1 are expressed in the uterine endometrium; however, data regarding their function are either very limited or conflicting. Understanding the role of GLUTs and hormonal mechanisms regulating their functions in the endometrium will lead us to a better competence in increasing fertility. It is, moreover, important to identify other GLUT in the endometrium that could function as compensatory transporters when some are inhibited or not functioning. Furthermore, a better understanding of endometrial GLUTs’ and SGLTs’ roles in early pregnancy may aid in the development of novel diagnostic markers and relevant therapies for miscarriage and other obstetrical complications. Understanding the mechanism linking the SGLTs and maternal obesity may help develop novel strategies to lower fetal overgrowth.

Endometrial cells undergo differentiation and maturation during the proliferative and mid-secretory phase of the menstrual cycle. These processes are crucial for the embryo-receptive state and thus for achieving and maintaining pregnancy, processes that depend on sufficient glucose intake, metabolism, and storage. Further studies are needed to determine the role of GLUTs in the endometrial stroma and epithelium. GLUT3, which is abundant in the endometrium and highly expressed in the placenta, and intracellular GLUT8 are interesting candidates for providing a mechanism in idiopathic infertility. Developing therapies affecting the glucose metabolism can also be potential aims.

Studies on glucose endometrial metabolism are necessary to understand the mechanism behind metabolism-related diseases affecting human reproductive health, such as preeclampsia, idiopathic infertility, and PCOS. Further clarification of GLUTs’ role in downstream signaling pathways governed by pregnancy-related hormonal changes could lead to the development of non-hormonal therapies for the prevention of pregnancy loss and implantation failures.

## Author Contributions

IVM designed the review, wrote the majority of the manuscript, performed revisions, and reviewed the complete manuscript. VK-K, JM, IŠ, and LŠ performed the literature search, wrote the manuscript and critically revised it. All authors contributed to the article and approved the submitted version.

## Conflict of Interest

The authors declare that the research was conducted in the absence of any commercial or financial relationships that could be construed as a potential conflict of interest.

## Publisher’s Note

All claims expressed in this article are solely those of the authors and do not necessarily represent those of their affiliated organizations, or those of the publisher, the editors and the reviewers. Any product that may be evaluated in this article, or claim that may be made by its manufacturer, is not guaranteed or endorsed by the publisher.
